# Morphologic Characterization of Trigeminothalamic Terminal Arbors Arising From the Principal Nucleus in the Macaque

**DOI:** 10.3389/fnana.2020.562673

**Published:** 2020-09-16

**Authors:** Dona Lee E. Andrew, Paul J. May, Susan Warren

**Affiliations:** ^1^Department of Occupational Therapy, University of Mississippi Medical Center, Jackson, MS, United States; ^2^Department of Neurobiology and Anatomical Sciences, University of Mississippi Medical Center, Jackson, MS, United States; ^3^Department of Ophthalmology, University of Mississippi Medical Center, Jackson, MS, United States; ^4^Department of Neurology, University of Mississippi Medical Center, Jackson, MS, United States

**Keywords:** somatosensory, trigeminal, thalamus, oral-facial, ventral posterior medial

## Abstract

The ventral posterior medial nucleus (VPM) is amandatory relay for orofacial sensory information targeting the primary somatosensory cortex. We characterized the morphology of VPM axons arising in the principal trigeminal sensory nucleus (pV) through injections of biotinylated dextran amine (BDA) placed in pV of *Macaca fascicularis* and* mulatta* monkeys. Labeled terminals formed a patchy bilateral distribution. Within contralateral VPM, patches were found primarily, but not exclusively, within the laterally located, vertical segment, and in ipsilateral VPM, primarily, but not exclusively, in the medially located, horizontal segment. Two fiber types were labeled: thin and thick. Thin fibers were poorly branched and diffusely distributed. They were studded with small *en passant* boutons. Most labeled fibers were thick and they branched extensively to form distinctive terminal arbors decorated with numerous boutons that varied in size and shape. Quantitative analysis of thick fiber arbor features showed little difference between the sides, although contralateral boutons were significantly larger than ipsilateral ones. Bouton distribution with respect to counterstained somata suggests that proximal dendrites are their main target. Indeed, ultrastructural examination demonstrated that they provide large diameter dendrites with numerous contacts. Direct comparison of thick fiber terminal arbors to cytochrome oxidase (CO) staining revealed that these arbors are much smaller than individual CO-rich patches believed to designate rods containing discrete body area representations. Thus, each terminal arbor appears to heavily innervate a small number of VPM neurons within a rod. This relationship would serve to maintain relatively small receptive fields within the topographic representation of the face.

## Introduction

The ventral posterior medial nucleus (VPM) is a critical relay for sensory signals arising from the face and the oral cavity. It receives ascending axons from neurons in the trigeminal sensory nuclear complex and its projection produces the face representation within the primary somatosensory (SI) cortex (Capra and Dessem, 1992; Cerkevich et al., 2013). Notably, the face representation is an area where the scaling factor between the receptor surface and the cortical representation is very high, similar to the hand representation (Sur et al., 1982). The structural characteristics of trigeminothalamic axons, as well as the dendritic geometry of VPM neurons, must allow the modality-specificity and topography of the facial representation to be preserved. At the same time, modest changes in receptive field properties and other characteristics can occur at this juncture (Veinante and Deschênes, 1999; Pierret et al., 2000; Friedberg et al., 2004). This study is aimed at providing a more detailed catalog of the morphologic features of the projections to VPM to help provide the structural basis for understanding the transfer and transformations of somatosensory information that occur at the thalamic level.

Previous studies of the trigeminal system have primarily examined the pathways serving the whisker and tooth representations, and most have used rodent or cat models (rat: Van der Loos, 1976; Williams et al., 1994; Veinante and Deschênes, 1999; Jacquin et al., 2015; cat: Sessle and Greenwood, 1976; Shigenaga et al., 1986; Yokota et al., 1986). In rats, for example, a point-to-point topography has been shown to exist between the representation of a single rat whisker and its corresponding thalamic barreloid. More generally, Peschanski (1984) reported that rat trigeminothalamic terminal arbors are restricted to spherical areas measuring approximately 100 μm in diameter. These axons exhibited numerous large, irregularly shaped boutons measuring up to 5 μm in diameter. We felt it would be useful to extend the examination of axonal morphology to a primate model to determine whether these morphological characteristics are generalizable to other species where the trigeminal system is not dominated by whisker afferents.

Early physiological investigations of the monkey VPM indicated the presence of both an ipsilateral and contralateral face representation (*M. mulatta*: Mountcastle and Henneman, 1952). Jones et al. (1986a,b) and Rausell and Jones (1991a) demonstrated that the monkey VPM can be partitioned into a laterally located vertical segment that mainly contains a representation of the contralateral face, and a medially located horizontal segment that contains a representation of the ipsilateral lips and intra-oral structures. Jones et al. (1986a) also utilized cytochrome oxidase (CO) staining to reveal a modular organization in both the vertical and horizontal segments of VPM consisting of CO-rich domains separated by the CO-poor matrix. The CO-rich domains form rods that extend along the anterior-posterior length of VPM. They suggested these CO-rich rods coincide with the patchy distribution of trigeminothalamic terminal fields labeled by anterograde transport of HRP from injections into the principal trigeminal nucleus (pV; Jones et al., 1986b; Rausell and Jones, 1991b). They further suggested that the CO-rich rods correlate with physiologically described rods within VPM that have homogeneous receptive field properties, i.e., they represent the same surface region and share the same modality. These rods are believed to project to focal regions (columns) within the primary somatosensory cortex (Jones et al., 1982). However, these earlier studies do not provide a description of individual pV trigeminothalamic axons within the primate VPM. More recently, cytochemical and morphological procedures have been combined to examine the possibility that trigeminothalamic terminals from pV express vesicular glutamate transporter isoforms (VGlut1 and VGlut2). These studies suggest that there may be subdivisions within the CO-rich rods (rat: Ge et al., 2010; macaque: Cerkevich et al., 2013).

The present study was undertaken to provide a detailed morphologic analysis of the trigeminothalamic projection within the VPM of macaque monkeys. We took advantage of the morphologic detail provided by the anterograde tracer, biotinylated dextran amine (BDA), to characterize the distribution and the morphology of terminal arbors within VPM that arise from pV. We directly compared the pattern of CO staining to the arrangement of BDA labeled trigeminothalamic axons in light of previous use of this approach to parcellate VPM. Finally, samples containing BDA labeled terminal arbors underwent electron microscopic analysis to extend the examination of trigeminothalamic boutons to the ultrastructural level and better identify their synaptic targets.

## Materials and Methods

All experiments were performed following NIH guidelines for animal care and use under protocols approved by the Institutional Animal Care and Use Committee (IACUC) at the University of Mississippi Medical Center [Institutional Assurance Number D16-00174 (A3275-01)]. Nine adult or young adult macaque monkeys [*Macaca fascicularis* (*N* = 6) and *Macaca mulatta* (*N* = 3)] of both sexes (three females and six males) were utilized in this study. Trigeminothalamic axons and their terminal arbors were labeled anterogradely from pressure injections of BDA (Molecular Probes, 10,000 MW) placed into pV in six animals. In three additional animals, which served as controls, injections of BDA were located in regions adjacent to the pV; i.e., immediately medial (*N* = 1), dorsal (*N* = 1), and lateral (*N* = 1) to the target nucleus.

### Surgical Procedures

Monkeys were sedated with an intramuscular dose of ketamine HCl (10 mg/kg), then anesthetized and maintained on inhalant anesthesia, Isoflurane (1–3%). Core body temperature and vital signs were recorded and maintained within normal ranges. Dexamethasone (1.0 mg/kg) was administered intravenously to control brain edema and a subcutaneous injection of atropine sulfate (0.05 mg/kg) was given to decrease tracheal secretions.

The animal’s head was placed in a stereotaxic frame (Kopf Instruments). Following a unilateral craniotomy, the dura mater was incised and reflected, and the parietal cortex between the central sulcus and the intraparietal sulcus was aspirated to reveal the anterior edge of the tentorium cerebelli. The tentorium was incised to visualize the junction of the dorsolateral surface of the cerebellum and the surface of the midbrain, then the two were gently separated. The point of exit of the trochlear nerve from between the cerebellum and the inferior colliculus (IC) was used as a landmark for the point along the middle cerebellar peduncle (MCP)/pons junction that lies over pV. The needle of a 1 μl Hamilton microsyringe containing the tracer was positioned at the peduncle-tegmentum junction and subsequently lowered 4.0–4.5 mm into pV. BDA was reconstituted in deionized H_2_O to give a final 10% solution. The total injection volume of BDA ranged from 0.1 to 0.5 μl. Several injections, covering a distance of 1.0 mm, were made along the trajectory of a single penetration, in an attempt to fill the pV. The incision was closed, and the animal was monitored during recovery. Animals received an intramuscular analgesic, buprenorphine (0.01 mg/kg) in the immediate post-operative period. In some cases, animals received additional injections of wheat germ agglutinin conjugated horseradish peroxidase (WGA-HRP) into facial and masticatory muscles to define trigeminal connections with brainstem motoneurons for a non-conflicting study.

Following a 14–21 day survival, animals were sedated with an intramuscular injection of ketamine HCl (10 mg/kg) and then received an intraperitoneal overdose of sodium pentobarbital (50 mg/kg), before being perfused transcardially. The perfusate consisted of a buffered saline prewash followed by a fixative solution containing 1.0% paraformaldehyde and 1.25–1.5% glutaraldehyde in 0.1M (pH 7.2) phosphate buffer (PB). Brains were blocked in the coronal plane and then post-fixed in the same fixative solution for at least 2 h, before being stored at 4°C in 0.1M (pH 7.2) PB.

### Histological and Ultrastructural Procedures

Coronal sections were cut at 100 μm using a vibratome (Leica VT1000). Sections were retained in serial order, and one or more one in three series were processed. Tissue sections were reacted using an Avidin-HRP procedure (May et al., 1997). Briefly, the tissue was first rinsed in 0.1 M (pH 7.2) PB containing Triton X-100 [0.05% for electron microscopy (EM) or 0.1% for light microscopy (LM)]. Next, sections were incubated overnight at 4°C in an Avidin-HRP solution (Vector Laboratories) diluted 1:500 in 0.1 M (pH 7.2) PB containing 0.05 or 0.1% Triton X-100. After rinsing with 0.1 M (pH 7.2) PB, the tissue was reacted in 0.05% diaminobenzidine (DAB; Sigma) containing 0.003% hydrogen peroxide and 0.01% nickel ammonium sulfate and cobalt chloride for 10–30 min. Sections were rinsed with 0.1 M (pH 7.2) PB. Individual sections were mounted on gelatinized slides, air dried, counterstained with cresyl violet, dehydrated, cleared and coverslipped.

In two animals, CO staining was performed on an adjacent series of thalamic sections to compare to the BDA results. They were reacted for CO activity using the method of Wong-Riley (1979). Briefly, sections were transferred to an incubation solution containing 0.06% diaminobenzidine (DAB), 0.06% cytochrome C (Sigma), and 4% sucrose in 0.1 M (pH 7.2) PB. Sections were then placed in the dark with gentle agitation at 37°C and examined periodically until the staining level was appropriate (3–5 h). Individual sections were mounted on gelatinized slides, air-dried, dehydrated, cleared, and coverslipped. In one additional animal, a double-label procedure to directly examine the relationship of the trigeminothalamic terminal arbors and CO-rich rod and CO-poor matrix compartments within VPM was employed. A separate series of 100 μm sections were used, in addition to the one processed just for BDA. First, the sections were reacted to reveal BDA, as described above. Next, sections were rinsed with 0.1 M (pH 7.2) PB and then reacted using the CO procedure.

The synaptic relationships between the BDA labeled trigeminothalamic boutons and VPM neurons were ultrastructurally investigated in two animals. Well labeled terminal arbors in both the contralateral and ipsilateral VPM were visualized in free-floating sections from an additional reacted series with a Wild M8 stereoscope, and samples containing them extracted. These tissue samples were processed using conventional EM techniques (Barnerssoi and May, 2016). Specifically, samples were fixed with 1.0% osmium tetroxide in 0.1 M (pH 7.2) PB, and then stained *en bloc* with 2.0% uranyl acetate. Following dehydration in a graded series of ethanols, they were infiltrated with EPON, and once embedded in the same, they were attached to EPON blocks. Semi-thin sections were taken to guide further trimming of the block. Then, ultrathin sections were obtained using an LKB diamond knife and mounted on copper mesh grids. Grids were stained with lead citrate. They were then viewed and photographed using a Zeiss EM 10C or Zeiss 906 transmission EM.

### Data Analysis

The distribution pattern of labeled trigeminothalamic terminal fields within the contralateral and ipsilateral VPM was plotted from a rostral-caudal series of sections by use of an Olympus, BH-2 microscope equipped with a drawing tube. For reference, drawings of the entire section were made using a Wild M8 stereoscope equipped with a drawing tube. The thalamic borders of VPM, its parvicellular subdivision (pc), the ventral posterior lateral nucleus (VPL), and centromedian nucleus (CM) were drawn based on published macaque brain atlases (Martin and Bowden, 2000; Saleem and Logothetis, 2007). We used the description of Jones et al. (1986a) to define the L-shaped VPM with a laterally located, vertical segment and medially located, horizontal segment, although no obvious architectural border between the two segments exists. Individual BDA labeled trigeminothalamic terminal arbors from throughout the region within VPM that contained label were drawn by use of a 100× oil objective on the Olympus, BH-2 microscope equipped with a drawing tube. In two examples, we serially reconstructed terminal arbors across multiple sections.

Also, the material was digitally photographed with a Nikon Eclipse 600 photomicroscope equipped with a Nikon DiS-R2 digital color camera by the use of Nikon Elements software. Images from multiple focal planes were combined to provide a focused view of the entire terminal arbor. Digitized images were adjusted for contrast and brightness in Photoshop.

Measurements of trigeminothalamic terminal arbor diameter and area were made using a morphometrics program [Microcomputer Imaging Device (MCID), Imaging Research Inc., St. Catharines, ON, Canada]. First, the outlines of well-isolated terminal arbors in single sections were drawn at a magnification of 600×. Samples from throughout the region containing label were taken. These terminal arbor outlines were digitized and used to calculate the arbor’s major axis diameter and area. Finally, we measured the size of the BDA labeled boutons produced by the labeled fibers. For each fiber type and each side, we photographed 12 well-isolated samples by use of the 100× objective. We collected a set of *z*-axis focal depths through the terminal arbor and merged them. We then used Image J to measure individual boutons in the image. While several measures were taken, we focused on the bouton area, because many boutons had complex shapes. Areas less than 0.1 μm^2^ were excluded as they likely represented incomplete examples. In all cases, measurements were compared using the student’s *t*-test, with significance set at *p* < 0.02.

## Results

The number of terminal arbors labeled varied between animals, but the areas of VPM in which they were distributed did not. In two animals, there were many terminal arbors labeled, but the terminal fields still did not fill the VPM target area, suggesting that not all pV axons were labeled. In two animals, there were moderate numbers of terminal arbors labeled, and in two animals only a handful of terminal arbors were labeled. In all cases, the injection site appeared to fill pV, so it is clear that the area of effective uptake was smaller than the area containing tracer reaction product. The injection sites differed in the density of the tracer and in the placement of the needle tract. There were fewer terminal arbors labeled when the tract was not centered in pV or the density of the tracer in the injection site was less. We have concentrated our LM analysis on the two best cases, and regard the others as confirmatory. There was no obvious pattern of differences observed between the male and female examples or the two macaque species.

### Distribution of BDA Labeled Trigeminothalamic Terminal Fields

**Figure 1:**
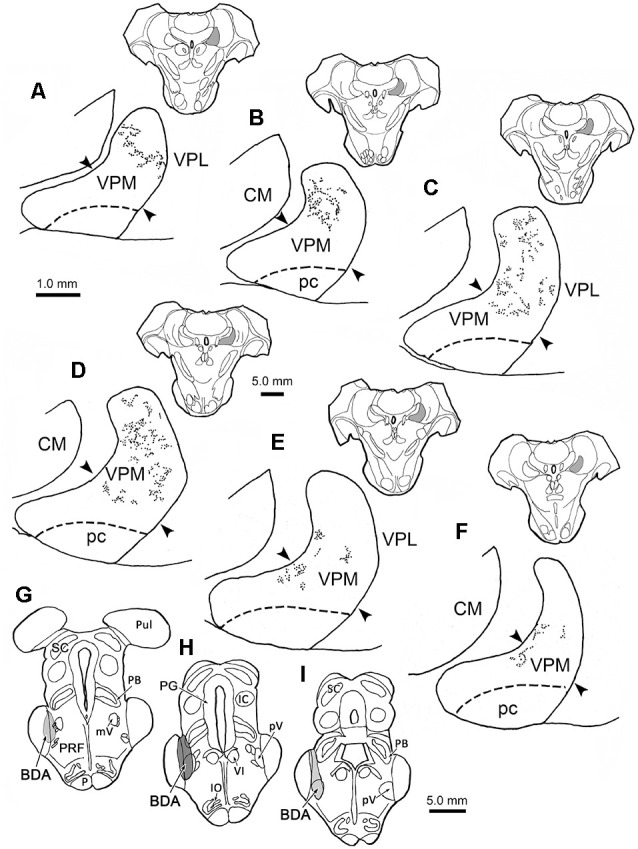
Contralateral terminal field distribution. The rostral **(A)** to caudal **(F)** distribution of labeled trigeminothalamic projections are plotted within the contralateral ventral posterior medial nucleus (VPM) following a biotinylated dextran amine (BDA) injection into pV **(G–I)**. The darker gray shading in **(H)** indicates the center of the injection site and the lighter shading the regions with less tracer **(G,I)**. BDA labeled terminal fields are distributed in a patchy fashion across the vertical segment of the nucleus. BDA labeled fields were also seen extending into the transition with the horizontal segment at more caudal levels **(D–F)**. The approximate location of the border between the horizontal and vertical segments is indicated by arrowheads. Inserts show the location of individual sections, which are spaced 500 μm apart. Shading in insert indicates the location of VPM in higher magnification views. Dorsal is always up in this and Figures 1–8.

Figure 1 shows the distribution of anterogradely labeled trigeminothalamic terminal fields within the contralateral VPM resulting from an injection of BDA into pV. This animal had the greatest degree of labeling of the six cases. The location and extent of the injection site are charted at three levels (Figures 1G–I). The injection site included all of pV, as well as portions of the adjacent MCP, pontine reticular formation, and the lateral edge of the parabrachial nucleus. As shown in this rostral (Figure 1A) to caudal (Figure 1F) series, the areas of label extended throughout the rostrocaudal dimension of the contralateral VPM, a distance of more than 3.0 mm. The BDA labeled terminal fields appeared as discrete patches embedded in regions that contained very few labeled terminals. A patch, as defined in the context of this study, refers to an area within the VPM containing a densely distributed trigeminothalamic terminal field. The contralateral terminal label was mainly distributed within the laterally located, vertical segment of the nucleus. Caudally, the trigeminothalamic terminal field pattern shifted ventromedially with some arbors extending into the region where the vertical and horizontal segments meet (arrowheads, Figures 1C,D). On entering the medially located, horizontal segment, the labeled terminal fields diminished and formed restricted patches along the dorsal edge of the nucleus (Figures 1E,F). The fact that the entire vertical segment was not filled with labeled terminals suggests that even though the injection site appeared to encompass all of pV, the effective area of uptake was considerably smaller. No anterograde label was observed in the adjacent CM or VPL (Figures 1A–F), or in the ventral posterior inferior nucleus, posterior nucleus, or anterior pulvinar (not shown). Labeled terminal arbors with homogeneously medium-sized boutons were observed in the intralaminar nuclei. The VPM parvicellular subdivision (pc; also termed VMb, Jones, 1985), which subserves taste, was also free of labeled terminals following this pV injection.

**Figure 2:**
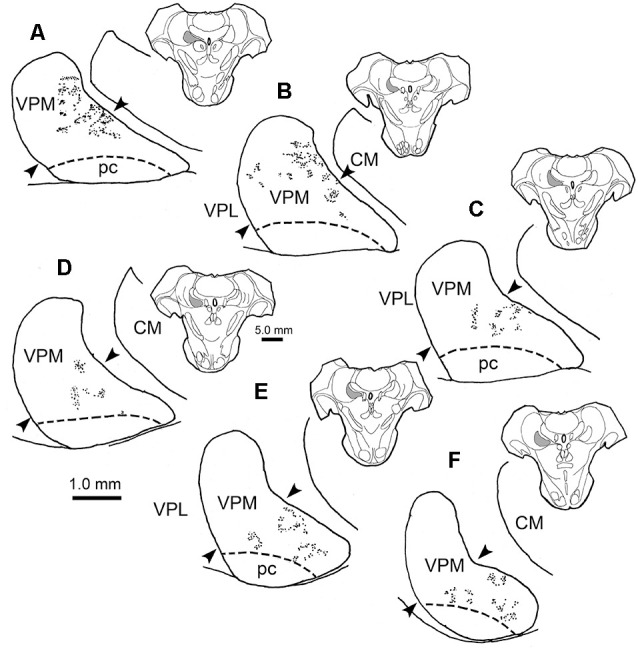
Ipsilateral terminal field distribution. The rostral **(A)** to caudal **(F)** patchy distribution of BDA labeled trigeminothalamic projections is plotted within the ipsilateral VPM following injection into pV (see Figures 1G–I). The majority of BDA labeled terminal patches are found in the horizontal segment of the nucleus at more caudal levels through the nucleus **(C–F)**. BDA terminal fields also extended up into the vertical segment of VPM at more rostral levels **(A–C)**. The approximate location of the border between the horizontal and vertical segments is indicated by arrowheads. Inserts show the location of individual sections, which are spaced 500 μm apart. Shading in insert indicates the location of VPM in higher magnification views.

Figure 2 charts the distribution of the anterogradely labeled trigeminothalamic terminal fields within ipsilateral VPM following the BDA injection shown in Figures 1G–I. The terminals were again distributed in patches. A substantial portion of these was located within the horizontal segment of the ipsilateral VPM (Figures 2C–F). However, rostrally (Figures 2A,B), BDA labeled terminal field patches were found in the medial part of the vertical segment. The distribution of terminal patches shifted in a ventromedial direction at successively more caudal levels, so that patches were confined to the horizontal segment at the caudal end of VPM (Figures 2C–F). Similar to the contralateral distribution pattern, no anterograde label was observed within pc or the thalamic nuclei adjacent to the ipsilateral VPM.

### Morphology of Trigeminothalamic Arbors in VPM

As shown in Figures 1, 2, BDA did not densely and continuously fill the vertical segment, contralaterally, or the horizontal segment ipsilaterally, unlike the pattern demonstrated with HRP transport previously (Rausell and Jones, 1991b). Instead, BDA labeled isolated patches within VPM. The size of the BDA labeled patches varied considerably, and the larger patches appeared to be made up of the terminal arbors of multiple axons. We concentrated our attention on the smaller patches that were more likely to derive from a single axon. As many of the characteristics of the ipsilateral and contralateral arbors are the same, we will describe them together in this section.

**Figure 3:**
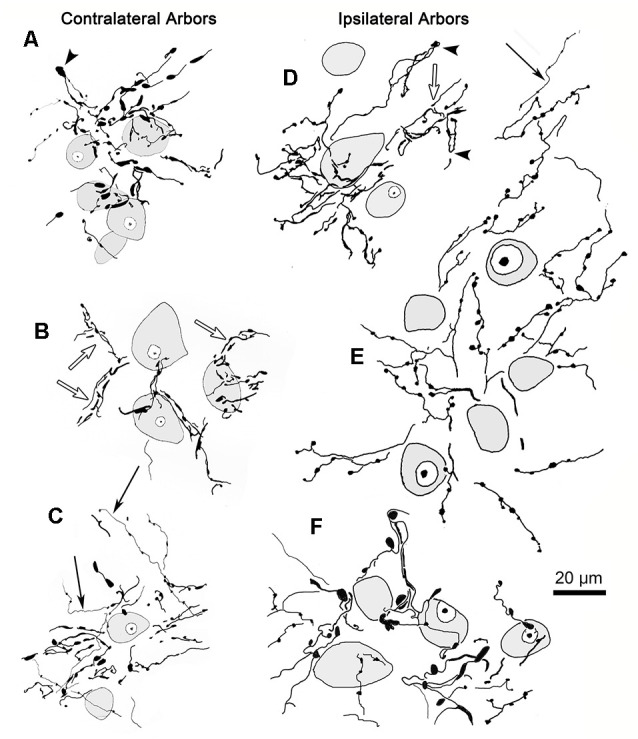
Labeled trigeminothalamic terminal arbor illustrations. Individual terminal arbors from the contralateral **(A–C)** and ipsilateral **(D–F)** VPM that were labeled following pV injections. These arbors are often organized around groups of counterstained somata (shading). They are produced by thick fibers covered with boutons. These boutons varied considerably in size and shape. Some axon segments displayed “hairpin” turns (arrowheads) at the periphery of the patch. The boutons were sometimes arranged in parallel, suggesting they terminated around proximal dendrites (open arrows). Note that for clarity not all labeled arbor elements are illustrated. The second type of labeled element, thin fibers (arrows), had long, undulating small-diameter axons studded with small, *en passant* boutons. Examples from two cases are included.

The characteristics of BDA labeled terminal arbors within smaller patches in the contralateral VPM are illustrated in Figures 3A–C and those in ipsilateral VPM are shown in Figures 3D–F. These examples are drawn from individual patches found in single 100 μm sections. Numerous segments of thick diameter fibers were found in each patch. It was difficult to follow the course of an individual labeled fiber for great distances within a patch due to their complex arrangement and convoluted course, which often extended beyond the depth of the section. The thick fibers often branched, and sometimes doubled back on themselves making “hairpin” turns at the periphery of the patch (arrowheads, Figure 3A contralateral, 3D ipsilateral). Each BDA labeled fiber was heavily decorated with numerous boutons. These boutons were primarily *en passant* in character. The bouton laden fibers were often concentrated in the neuropil immediately surrounding counterstained thalamic neuron somata (shaded profiles) and sometimes appeared to radiate out from these cells as if following their dendrites (open arrows, Figure 3B contralateral, 3D, ipsilateral). However, they were rarely directly opposed to somata.

Thin, undulating BDA labeled fibers were also found in VPM. These were fewer in number than thick fibers. They were rarely seen to branch and were not observed branching from thick fibers. While these thin fibers sometimes entered an arbor formed by a thick fiber (arrows, Figure 3C contralateral, 3E ipsilateral), they were more likely to be located between them. Thin fibers could be followed for considerable distances as they traversed the neuropil found in areas without labeled thick fiber arbors. When present near a patch, their relationship was variable; sometimes passing through the arbor or along its fringes. Thus, their association with the thick fibers appears to be random. Thin fibers displayed multiple, small boutons that were oval in shape and *en passant* in location. In contrast to thick fiber boutons, these boutons were quite homogeneous in composition. There was no evidence suggesting a synaptic relationship between the thin fibers and any specific element in the neuropil.

**Figure 4:**
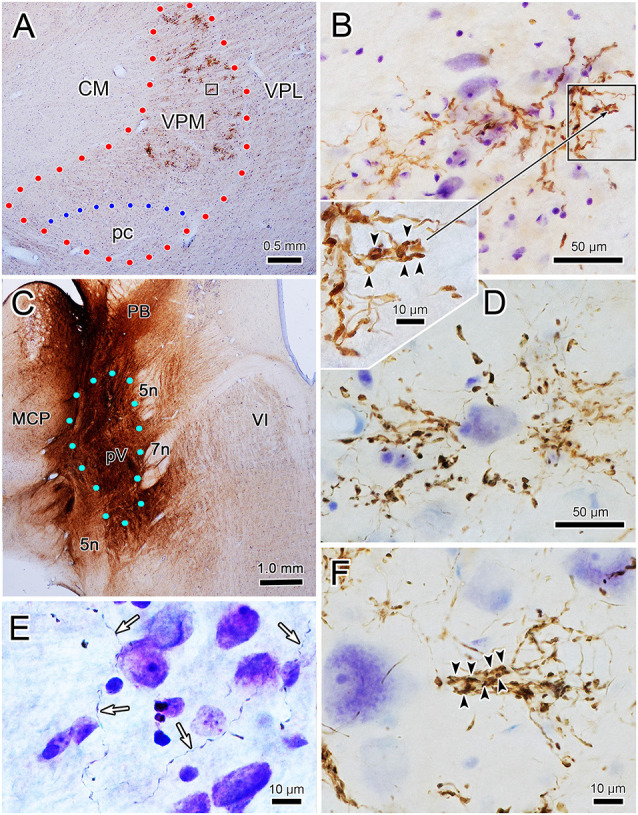
Contralateral terminal arbor morphology. Panel **(A)** is a low power view of the contralateral labeled terminal fields. The injection site that produced the label seen in panel **(A)** (and in Figures 1, 2) is shown in panel **(C)**. Examples of BDA labeled terminal arbors located within the contralateral VPM from two different cases are shown (see Figures 6C–E for second injection site). The boxed region in panel **(A)** is shown at higher magnification in panel **(B)**. A portion of this arbor is show at even higher magnification in the adjacent inset. Arrowheads indicate boutons lined up in a manner suggesting they surround a dendrite. A similar organization is seen in panel **(F)**. Plates **(B,D,F)** are examples of arbors produced by thick fibers. Examples of thin fibers indicated by white arrows are shown in **(E)**. Blue dots indicate pV boundary in panel **(C)**.

**Figure 5:**
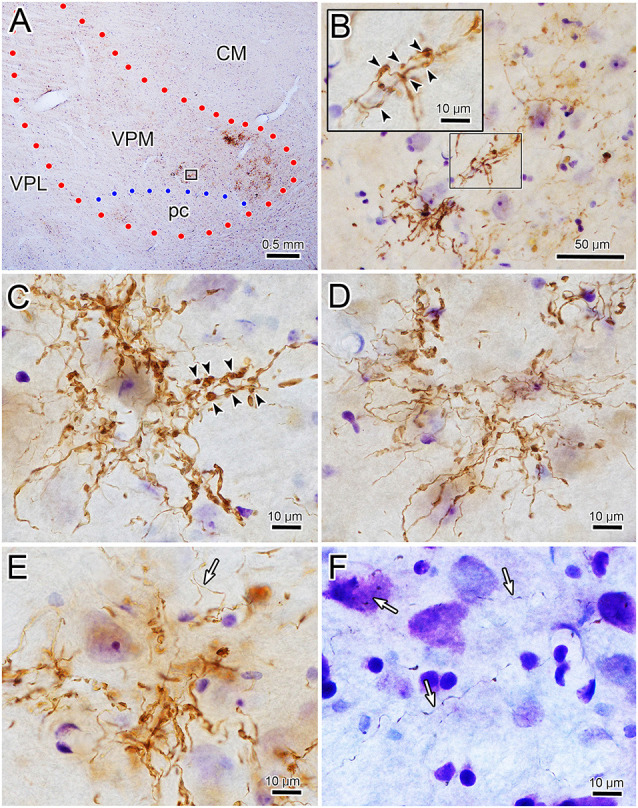
Ipsilateral terminal arbor morphology. Panel **(A)** is a low power view of the pattern of ipsilateral VPM terminal fields labeled from the injection site shown in Figure 4B. Panels **(B–E)** are examples of the labeled thick fiber arbors from this case and that shown in Figures 6C–E. The boxed region in panel **(A)** is shown at higher magnification in panel **(B)**. Details of a portion of this terminal arbor are shown in the insert where boutons appear to be arranged around a dendrite (arrowheads). A similar arrangement is seen in panel **(C)**. Thin fibers (white arrows) pass near **(E)** and in regions between **(F)** the thick fiber patches.

The core of the injection site from the case illustrated in Figures 1, 2 is further demonstrated in Figure 4C. The labeled terminal field that resulted from this injection is distributed contralaterally within the laterally located, vertical segment of VPM, as shown in Figure 4A, and distributed ipsilaterally in the medially located, horizontal segment, as shown in Figure 5A. Photomicrographs of individual BDA labeled terminal arbors within contralateral VPM are shown in Figures 4B,D,F, and those within the ipsilateral VPM are shown in Figures 5B–E. The thick fibers on both sides were decorated with both *en passant* and terminal boutons. Note the variation in size of the boutons within arbors. While there was little evidence of close associations between these boutons and the somata of counterstained neurons, some labeled arbors formed a three-dimensional complex whose arrangement suggested they extend along the proximal dendrites of individual thalamic cells (Figures 4B,F contralaterally, Figures 5B,C ipsilaterally). For example, the terminal arbor enclosed by the box in Figure 4A is shown at higher magnification in Figure 4B. The insert in the lower corner shows axons laden with numerous boutons of varying sizes. In examples like this one, the boutons display parallel rows, as if associating with either side of a dendrite (arrowheads, inset). Figure 4F shows another example where a portion of the terminal arbor extends in a manner that suggests it is following the proximal dendrite of the cell whose soma is seen on the left. The arrowheads again indicate how the boutons appear to encompass this unstained dendrite. Similar examples from the ipsilateral side are shown in Figure 5. The terminal arbor enclosed by the box in Figure 5A is shown at higher magnification in Figure 5B, and the insert shows an area in which the boutons form parallel rows (arrowheads), presumably around an unlabeled dendrite. Another example of this arrangement is shown in Figure 5C. Examples of undulating thin fibers (white arrows) can also be observed in contralateral (Figure 4E) and ipsilateral (Figures 5E,F) VPM. These thin fibers rarely branched and displayed much smaller boutons. Some examples were located near thick fiber terminal arbors (Figure 5E). However, thin fibers were more commonly observed in the areas of neuropil that lay outside the thick fiber patches (Figures 4E, 5F).

**Figure 6:**
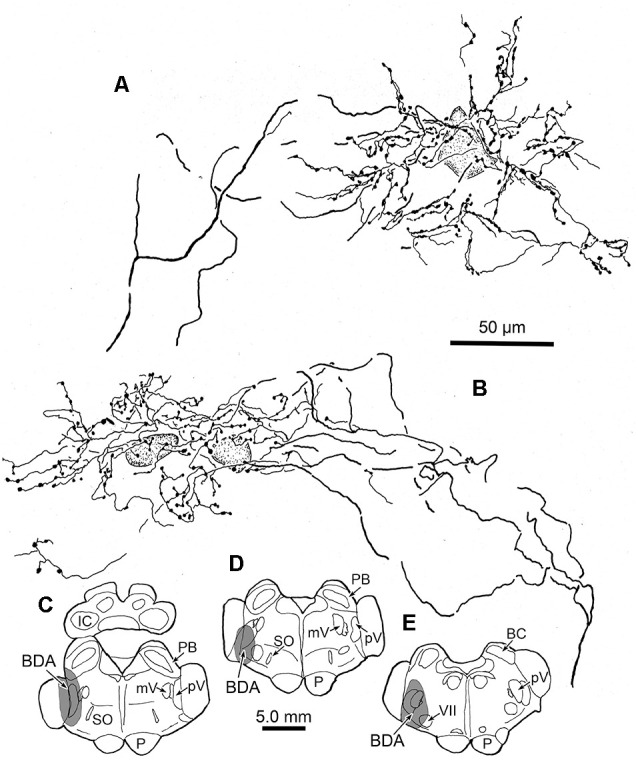
Serial reconstructions of BDA labeled arbors. A contralateral **(A)** and an ipsilateral **(B)** trigeminothalamic terminal arbor from a second case with a BDA injection (stipple) placed into pV **(C–E)** provide detailed insight into their organization. These were reconstructed from multiple adjacent sections by use of a drawing tube. The parent fiber (arrow) divides into several branches, which then converge to form the terminal arbors that are associated with counterstained somata (stipple).

We wished to gather a more complete picture of the features of the thick trigeminothalamic arbors found in VPM, so we serially reconstructed an example from each side. Figure 6 shows these serial reconstructions of a contralateral (A) and ipsilateral (B) trigeminothalamic terminal arbor. These arbors are taken from a second animal whose pV injection is shown (Figures 6C–E). In each case, parent axons (arrows, Figures 6A,B) approach the medial border of the nucleus and subsequently divide into several branches, which then converge to form a single patch within VPM. The contralateral arbor (Figure 6A) is more clearly arranged around the dendrites of a single thalamic neuron (stippled profile). The ipsilateral field arborizes around a small group of cells (stippled profiles), without apparent regard to the neuronal dendrites. These thicker fibers were decorated with numerous boutons. Both terminal arbors were approximately 150 μm wide. They extended within three adjacent sections, so they reached almost 300 μms in the rostrocaudal dimension.

### Quantitative Analysis

Well defined terminal arbors like those shown in Figures 3–5, were chosen for quantitative evaluation of the axonal field morphology in the two animals that had the most arbors labeled (see Figures 1G–I, 6C–E for injection sites). BDA labeled terminal arbors were sampled from both the contralateral VPM (*n* = 58) and ipsilateral VPM (*n* = 54). Individual arbor diameter measurements were grouped into 25 μm bins for analysis. The contralateral VPM arbor diameters ranged between 25–175 μm, with a mean of 86 (SD ± 31) μm, while those in ipsilateral VPM ranged between 75 and 200 μm, with a mean of 99 (SD ± 26) μm. Considerable overlap in the ranges of diameter between the two groups of arbors exists, as 88% of the contralateral and 94% of the ipsilateral terminal arbors had diameters ranging from 75 to 150 μm, and their distributions were not significantly different from each other (*p* = 0.907). While the techniques we employed did not allow us to determine whether the labeled arbors used in this analysis always represent the arbor of a single trigeminothalamic axon, their relatively small size and the fact they had dimensions similar to our serially reconstructed examples and those of intra-axonally stained examples in other studies (Veinante and Deschênes, 1999) suggests that this was often the case.

The trigeminothalamic axonal arbor area was calculated for this sample. Individual arbor area measurements were grouped into 5,000 μm^2^ bins for analysis. The area occupied by BDA labeled, contralateral terminal arbors ranged from 5,000 to 30,000 μm^2^, while the ipsilateral terminal arbor areas ranged from 5,000 to 25,000 μm^2^. Their mean areas were 11,595 (SD ± 6,504) μm^2^ for the contralateral arbors and 11,456 (SD ± 4,324) μm^2^ for the ipsilateral arbors. Based on these measurements, the ipsilateral fields and the contralateral fields had virtually the same areas (*p* = 0.904).

Measurements of the labeled boutons of the thick fiber arbors (injection site in Figure 1) revealed that those on the contralateral side were somewhat larger (mean = 3.55 μm^2^, *SD* = 2.67 μm^2^, median = 2.90 μm^2^, range = 0.12–15.43 μm^2^) than those on the ipsilateral side (mean = 2.57 μm^2^, *SD* = 2.13 μm^2^, median = 2.13 μm^2^, range = 0.10–13.04 μm^2^). This is primarily due to a larger percentage of the ipsilateral boutons having areas smaller than 2 μm^2^. The difference between the contralateral and ipsilateral bouton areas is significant (*p* ≪ 0.02). While the contralateral boutons may be larger, it appeared that there were about half as many boutons in the contralateral arbors, for we measured 760 boutons on the contralateral side and 1,396 on ipsilateral side in the 12 images we took of each. We did not seen a significant difference (*p* = 0.33) in the size of the boutons located on thin fibers of the contralateral side (mean = 0.58 μm^2^, *SD* = 0.58 μm^2^, median 0.39 μm^2^, range = 0.10–6.69 μm^2^, *n* = 341) compared to those on the ipsilateral side (mean = 0.51 μm^2^, *SD* = 0.48 μm^2^, range = 0.10–3.38 μm^2^, *n* = 457). Since 85% of the ipsilateral and 86% contralateral boutons were less than 1.0 μm^2^, and 13% and 12%, respectively, were between 1.0 and 2.0 μm^2^ in area, they were, on average, far smaller than most of either the ipsilateral or contralateral thick fiber boutons (*p* ≪ 0.02). Comparison of the ranges of the thin and thick fiber boutons indicates there is overlap, so, possibly, a portion of the small boutons (<2 μm^2^) measured in the thick fiber arbors may have belonged to thin fibers.

### Relationship of Labeled Terminal Arbors and CO-Rich Rods

**Figure 7:**
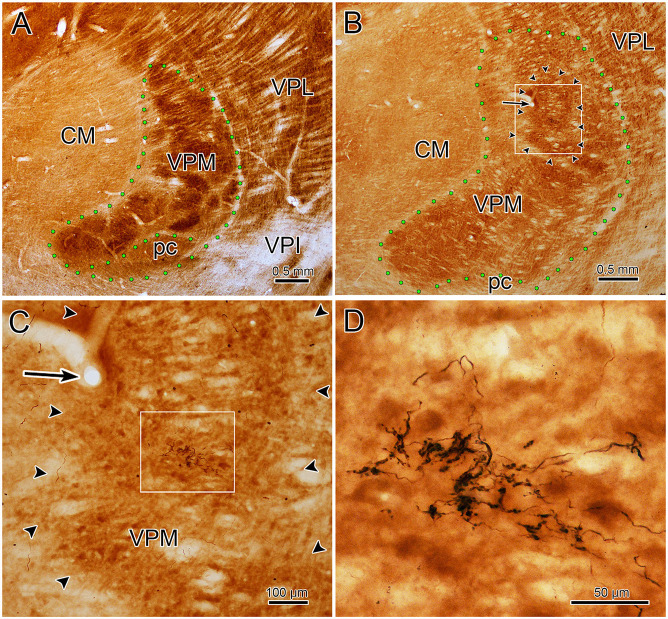
Contralateral arbors in cytochrome oxidase (CO)-rich rods. The relationship of BDA labeled trigeminothalamic terminal arbors to CO-rich rods in contralateral VPM is shown (see Figures 6C–E for the injection site.) Panel **(A)** is a low power photomicrograph of just the CO labeling pattern within the contralateral VPM, showing the dark rods and light septa. Panel **(B)** shows the contralateral VPM more lightly labeled for CO, but with dual-labeling for BDA labeled fibers. Arrowheads outline a rod contained in the box shown at higher magnification in panel **(C)** (arrows show the corresponding blood vessel). The border between this rod and an adjacent one is easily seen on the left side of the plate by the difference in CO labeling intensity. A labeled trigeminothalamic terminal arbor crosses less than a third of the CO-rich rod. Green dots indicate VPM and pc boundaries in panels **(A,B)**. The boxed region in panel **(C)** is shown at higher magnification in panel **(D)** to demonstrate the terminal arbor.

**Figure 8:**
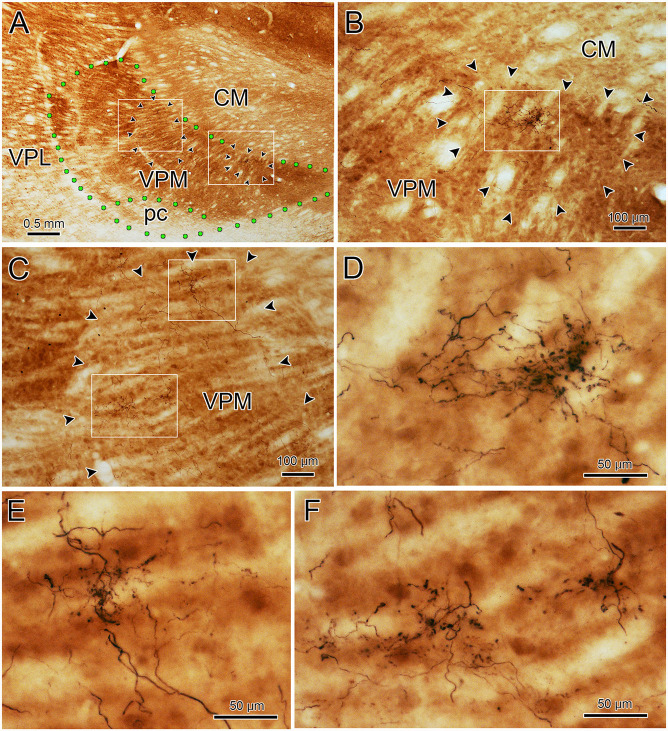
Ipsilateral arbors in CO-rich rods. The relationship of individual BDA labeled trigeminothalamic terminal arbors to CO-rich rods in ipsilateral VPM (see Figures 6C–E for the injection site). Panel **(A)** is a low power photomicrograph of the CO-rich labeling pattern within the ipsilateral VPM. Arrowheads outline two CO positive rods. As indicated by the boxes in panel **(A)**, these rods are shown at higher magnification in panels **(B,C)**. The more medial rod **(B)** contains a single BDA labeled terminal arbor that is less than half the diameter of the rod (arrowheads). The boxed region is shown at higher magnification in panel **(D)** to demonstrate the structure of the arborization. Panel **(C)** contains a larger, more lateral CO-rich rod, which contains two terminal arbors. The boxed regions containing the BDA labeled arbors are shown at higher magnification in panel **(E)** (dorsal box) and **(F)** (ventral box). They arborize within only a small region of the rod. Green dots indicate VPM and pc boundaries in **(A)**.

To assess the relationship between the CO compartments and trigeminothalamic terminal arbors, sections adjacent to those reacted for BDA were processed to reveal the presence of CO activity. Figure 7A is a coronal section through VPM demonstrating CO-rich domains separated by thin septa of CO-poor matrix. The CO-rich domains, termed rods, are thought to correlate with physiologically defined rods that display homogeneous response characteristics (Jones et al., 1982; Rausell and Jones, 1991a). We measured 60 rods from material like this. The long axis diameter of the rods ranged from 70 to 440 μm, with a mean diameter of 180 μm (SD ± 87.27). This is considerably larger than the average labeled terminal arbor diameter (86 μm contralateral, 99 μm ipsilateral), so we directly compared the labeled axons to the pattern of CO staining in double-labeled material. A CO-rich rod outlined by arrowheads is shown in Figure 7B. The individual contralateral BDA labeled terminal arbor it contains fails to fill the entirety of the CO-rich rod (box, Figure 7C). A higher magnification image of the individual arbor is shown in Figure 7D. Figure 8A is a coronal section through the ipsilateral VPM stained to demonstrate CO-rich rods, two of which are outlined by arrowheads. The CO-rich rod in Figure 8B contains a single terminal arbor shown at higher magnification in Figure 8D. Even though this is a relatively small rod, the labeled arbor does not reach across it. The large CO-rich rod in Figure 8C (arrowheads) contains two terminal arbors (boxes, Figure 8C). The arbors (shown at higher magnification in Figures 8E,F) are separated by an area containing no labeled axons. Thus, in both contralateral and ipsilateral VPM cases, the BDA labeled terminal arbors were constrained within, but did not fill, the CO-rich rods.

### Ultrastructure of pV Trigeminothalamic Terminals

**Figure 9:**
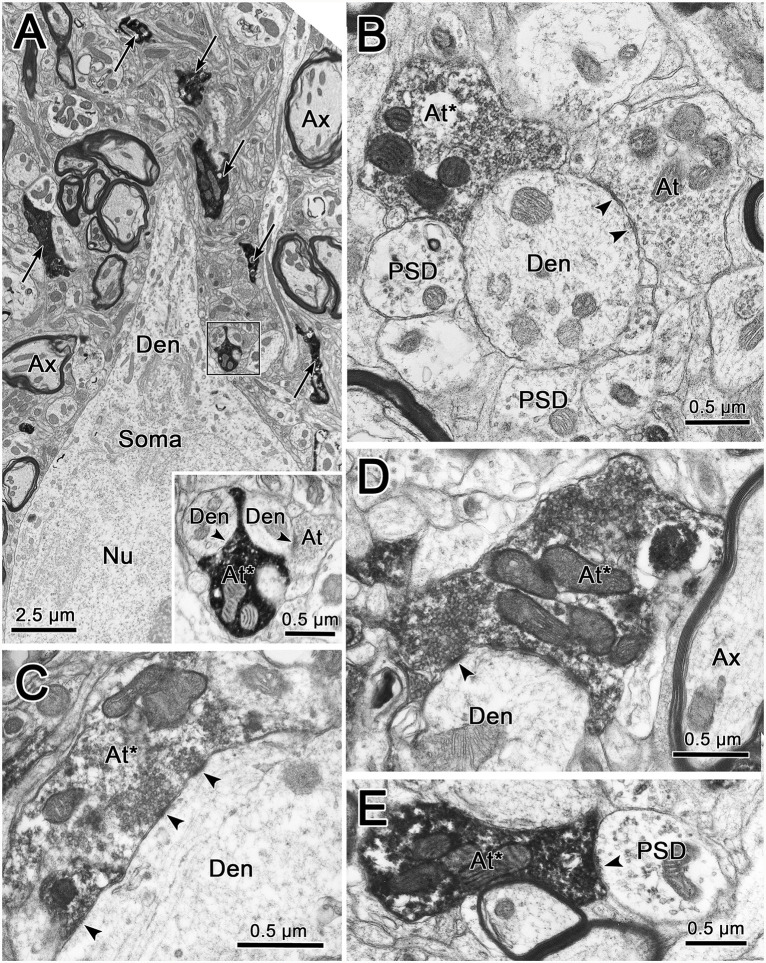
Ultrastructure of labeled trigeminothalamic terminals. Panel **(A)** is a low magnification image of a thalamic neuronal soma and proximal dendrite (Den). Note the indented membrane of the nucleus (Nu). The surrounding neuropil contains myelinated axons (Ax) and electron-dense labeled axon terminals (arrows). One example (boxed region) is shown at higher magnification in the inset. Labeled axon terminals (At*) are shown synaptically contacting (arrowhead) conventional dendrites in panels **(B–D)**. In panel **(C)**, where the cut through the dendrite is closer to longitudinal, multiple synaptic contacts by a lightly labeled terminal can be observed. In some cases, presynaptic dendrites (PSDs) were also associated with the labeled terminals **(B,E)**.

The ultrastructural characteristics of the BDA labeled trigeminothalamic profiles (*n* = 74) from thick fiber terminal arbors were examined qualitatively. As no obvious difference was seen between the contralateral and ipsilateral samples concerning vesicle type, synaptic densities, or target profiles, they will be described without attribution. Figure 9 shows examples of BDA labeled trigeminothalamic terminal associations within VPM. The samples contained 20–30 μm diameter somata whose euchromatic nuclei displayed infoldings (Figure 9A). Electron dense, BDA labeled synaptic terminals (arrows) were often located near their proximal dendrites (Den). In many cases, the reaction product in the labeled axon terminal (At*) was so electron-dense, that the ultrastructural characteristics of the axon terminal were obscured (Figure 9A insert, **E**). In more lightly labeled cases, clear spherical vesicles could be observed (Figures 9B–D), although the reaction product was still clearly present, by comparison to unlabeled synaptic terminals (At; Figure 9A insert, **B**). Slightly asymmetric synaptic densities (arrowheads) were seen between labeled terminals and the dendrites (Figures 9C–E). Larger synaptic terminals often displayed multiple contacts (Figure 9C). Presynaptic dendrites (PSD) that contained scattered vesicles were also present in the neuropil of the VPM (Figures 9B,E). While these were also associated with the BDA labeled terminals, only rarely was there any evidence of synaptic contact between the two (Figure 9E). Axo-somatic contacts were not a common finding in our samples.

**Figure 10:**
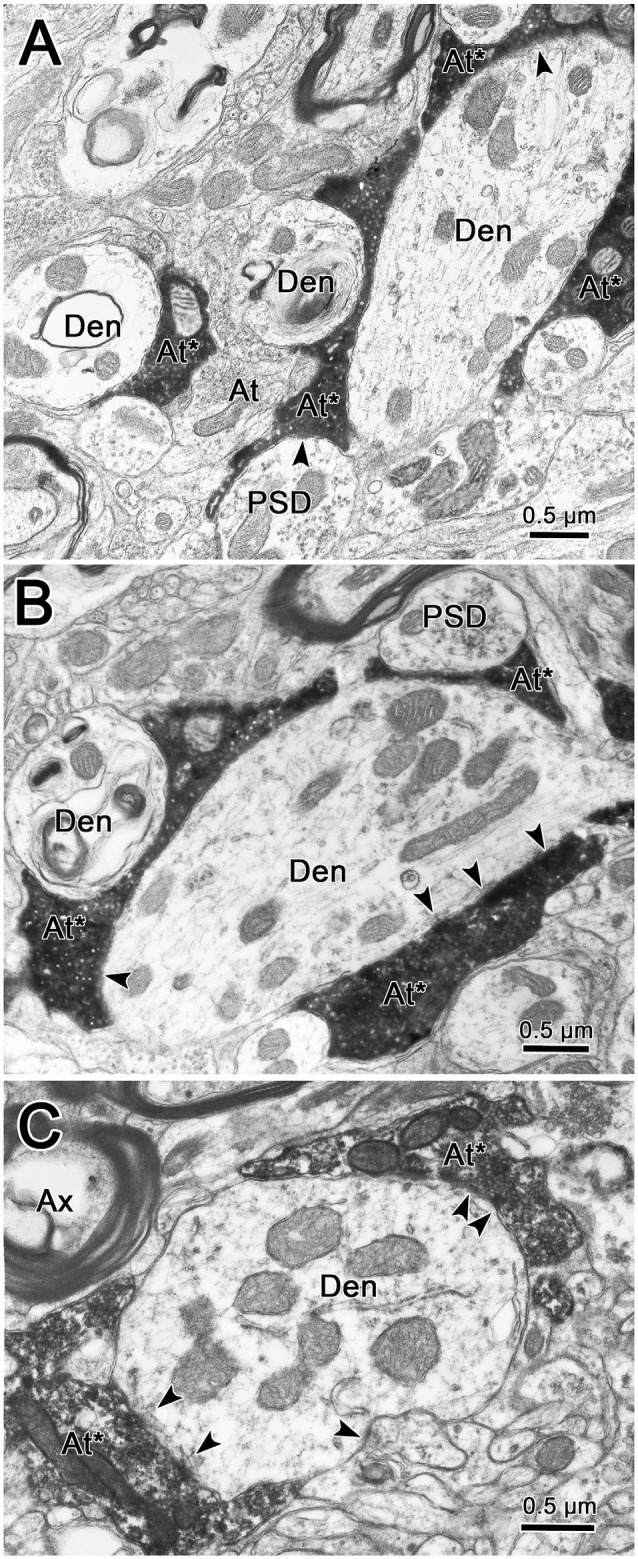
Labeled trigeminothalamic terminals contacting proximal dendrites. Panels **(A,B)** are semi-serial sections through a large dendrite associated with labeled axon terminals (At*). Multiple synaptic contacts (arrowheads) from multiple large terminals are present. Note the scalloped shape produced by cupping adjacent smaller conventional dendrites and a PSD. Panel **(C)** shows another example of multiple labeled synapses contacting a large proximal dendrite.

As shown in Figures 9B–D, labeled terminals contacted dendrites with a variety of diameters, including smaller dendrites with diameters less than 1.0 μm. This likely correlates with their widespread location on the dendritic tree. However, the most common relationship between labeled synaptic terminals and postsynaptic elements is shown in Figure 10. In these cases, presumably proximal, medium to large (1–3 μm diameter) dendrites were observed to be postsynaptic to numerous BDA labeled terminal profiles (At*). The large BDA labeled terminals often displayed scalloped borders due to the fact they cupped the elements in the neuropil (Figure 9A insert; Figures 10A,B). The presence of multiple labeled synaptic terminal profiles contacting the same dendrite is likely the ultrastructural correlate of the parallel boutonal arrangements observed at the LM level (arrowheads; Figures 4B,F, 5B,C).

## Discussion

This report is the first primate study to provide a detailed description of the morphologic and ultrastructural characteristics of the ipsilateral and contralateral trigeminothalamic terminal arbors originating in pV. The results support previous work showing the vertical segment primarily contains contralaterally projecting pV terminals, and the horizontal segment primarily contains the ipsilateral pV projection. However, we observed more overlap between contralateral and ipsilateral projections than previous studies. The labeled pV axons formed discrete terminal arbors in VPM that consisted of thick fibers with dense *en passant* and terminal boutons of a wide variety of sizes and shapes. A comparison of the thick fiber terminal arbors to the pattern of CO staining revealed that they are restricted to smaller subdomains within the CO-rich rods. The thick fiber arbor size and the arrangement of their boutons seen at the LM and EM level suggests individual axons strongly influence a few cells in a rod by terminating extensively on their proximal dendrites. A second labeled component, consisting of thin fibers studded with small *en passant* swellings, displayed no strict relationship to the patches formed by the thick fibers, so they presumably serve a different, less topographically specific, function.

### Technical Considerations

In experiments of this type, injection site spread is a potential contributor to the distribution of terminal label. In the present study, the brainstem structures adjacent to pV were involved, to varying degrees, by the spread of the tracer. However, control injections that lay immediately lateral to pV in the MCP, or lay medial to pV and involved the lateral edge of the pontine reticular formation, or dorsal to pV and involved the parabrachial nucleus (PBN) did not produce VPM labeling. The rostral part of the spinal trigeminal nucleus pars oralis (sVo) was involved in the injection site. So, possibly some of the terminal fields in VPM originated from neurons in sVo, as this nucleus is reported to provide input to VPM (cat: Yasui et al., 1983; Shigenaga et al., 1986; rat: Chiaia et al., 1991; de Chazeron et al., 2004). However, sVo projects preferentially to the posterior nucleus in rodents (Diamond et al., 1992), and we did not see labeled fibers in this nucleus, suggesting we did not have a major involvement of sVo.

The PBN has been reported to be a source of afferent input to VPM in the rat (Fulwiler and Saper, 1984; Krukoff et al., 1993). Rat PBN neurons have been shown to project to pc, the taste region of the thalamus (Fulwiler and Saper, 1984; Krukoff et al., 1993). In the macaque, tritiated amino injections into PBN failed to label pc but, instead labeled terminals more anteriorly in ipsilateral VPM (Pritchard et al., 2000). They suggested that this projection was related to visceral sensations, not taste. In the present study, no evidence of anterograde label in the taste subdivision, pc, was seen. The spread of BDA into lateral PBN could have contributed terminal fields in ipsilateral VPM, although our control injection in lateral PBN did not produce VPM labeling.

Based on comparisons with primary afferent morphology, one might expect that the thin fibers we observed convey noxious modalities. Both pV and sVo contain polymodal cells that respond to both non-noxious and noxious stimuli (Yu and King, 1974; Azerad et al., 1982; Dallel et al., 1990). Thus, on physiologic grounds, both pV and sVo might be expected to be the source of a thin fiber projection. Previous reports have suggested that spinal trigeminal projections are more diffuse, and are concentrated within non-barreloid areas of VPM, ventral and caudal to the area containing dense inputs from pV (monkey: Rausell and Jones, 1991b, rat: Peschanski, 1984; Williams et al., 1994; Veinante et al., 2000). The pattern of thin fiber distribution in the present study is not unlike this. Consequently, it is also possible that these thinner fibers represent trigeminothalamic axons from the spinal nucleus, which were labeled *via* their collateral branches to pV (Ikeda et al., 1984; Warren and May, 2013). Alternatively, collateral labeling of non-specific axons (e.g., locus coeruleus) that may supply both the trigeminal nuclei and VPM is a possibility.

### Distribution of Label in VPM and Relevance of the Ipsilateral Projection

The origin of the ipsilateral pathway to VPM has been described in the monkey and cat as arising from neurons confined to the dorsomedial region of pV (Burton and Craig, 1979). The dorsomedial region of pV receives primary afferents mainly from the mandibular branch of the trigeminal nerve, along with proprioceptive fibers from the jaw and the teeth, by way of the central projections of mesencephalic trigeminal neurons (Capra and Dessem, 1992). Thus, in the rhesus monkey, the dorsal trigeminothalamic projection, which primarily targets ipsilateral VPM, is thought to arise mainly from receptive fields found on lower lip and teeth, tongue, and oral cavity (Mountcastle and Henneman, 1952; Jones et al., 1986b; Rausell and Jones, 1991a). Presumably, it is information from this pathway that is provided to VPM by the terminal arbors we observed in the horizontal segment.

The present study confirms previous reports examining the contralateral and ipsilateral trigeminothalamic projections to VPM in the cat (Matsushita et al., 1982; Yasui et al., 1983) and in the primate (Burton and Craig, 1979; Ganchrow and Mehler, 1986). In agreement with the data from HRP injections in macaque pV (Jones et al., 1986b), the contralateral terminal fields were concentrated in the vertical segment of VPM, and the ipsilateral terminal fields were concentrated in the horizontal segment. This suggests that each pathway mainly occupies adjacent, non-overlapping territories. However, we also observed a limited overlap between the ipsilateral and contralateral projections. This suggests a possible convergence of these inputs onto a portion of the thalamocortical cells. This limited overlap could contribute to a small number of VPM neurons (10%) which are reported to display bilateral receptive fields (*M. fascicularis*: Bushnell and Duncan, 1987).

Most reports describing the existence of an ipsilateral intraoral representation in the primate VPM have described this representation as small (*M. mulatta*: Mountcastle and Henneman, 1952; squirrel monkey (*Saimiri*): Bombardieri et al., 1975; spider monkey (*Ateles*): Pubols, 1968). For example, Bushnell and Duncan (1987), recording in an alert rhesus monkey, found only 10% of isolated VPM neurons displayed either ipsilateral (5.6%) or bilateral (4.5%) intraoral receptive fields. However, their recordings focused on the vertical segment of VPM. In contrast, the size of the contra- and ipsilateral projections was about the same in our animals. This is as per physiological recordings that indicated 40% of VPM neurons in macaques have ipsilateral intraoral receptive fields (Jones et al., 1986b).

The large ipsilateral intraoral face representation is recapitulated in the face representation in area 3b, which has a substantial ipsilateral intraoral representation (Manger et al., 1996). Furthermore, Cerkevich et al. (2013) determined that neurons in the macaque lateral VPM (vertical segment) were retrogradely labeled from injections made into area 3b regions that contain the representation of the contralateral face, chin and upper lip. In contrast, retrogradely labeled neurons within the medial VPM (horizontal segment) were labeled from injections of area 3b that contained the representation of ipsilateral intraoral regions. These distributions agree with the main distributions of contralateral and ipsilateral terminals in VPM observed in the present study. Of note, the tongue representation included cells with ipsilateral, contralateral, and bilateral receptive fields. This may account for areas near the border between the horizontal and vertical segments where we saw both contralateral and ipsilateral terminals.

### pV Terminal Arbors and VPM Rods

One of the striking characteristics of the pV terminal fields in VPM was its patchy nature. This patchy characteristic has been described by several investigations of the rat thalamus. Peschanski (1984) reported that regardless of their origin in the trigeminal sensory complex, the terminal fields resembled “bushy arbors.” Chiaia et al. (1991) reported that the labeling pattern in the rat VPM following injections of WGA-HRP into pV was patch-like. Williams et al. (1994) and others (Veinante and Deschênes, 1999; Veinante et al., 2000; Mo et al., 2017) noted that intra-axonal staining of physiologically identified trigeminothalamic axons produced terminal arbors, measuring 60–100 μm in diameter and restricted to single VPM barreloids. All the terminal arbors described above resembled those shown here, concerning size and general shape.

Physiological studies have indicated that both VPL and VPM are organized into rods of thalamocortical neurons with similar properties that extend in the anterior-posterior direction (primate: Jones et al., 1982; Jones, 1983; cat: Rainey and Jones, 1983). All the cells in a given thalamic rod respond to stimulation of the same region on the body or face. Furthermore, following injections of HRP into individual medial lemniscal axons, it was shown that a single axon’s terminal arbor ranged from 700 to 1,200 μm in the sagittal plane of VPL (Jones, 1983; Rainey and Jones, 1983), but was restricted in other axes. These axons gave rise to more than one focal terminal arborization, giving them the appearance of a “grape arbor” in the sagittal plane. Viewed in frontal sections, the VPL arbors have very similar morphologies to those observed in the present study (Jones, 1983). In our material, a few large patches in both the contralateral and ipsilateral VPM could be followed through sections spanning 900 μm. This suggests that both ventral posterior systems that convey “lemniscal” sensory information utilize a similar rod-shaped arbor configuration. It seems likely that the terminal arbors observed in the present study are associated with physiologically homogeneous rods in the primate VPM. However, it should be noted that the VPM terminal arbors had smaller diameters in the coronal plane (less than 200 μm) than medial lemniscus arbors (250–500 μm). This suggests that the representation of the face may have a finer grain of resolution within VPM than the body representation has within VPL. The serial reconstructions (Figure 6) indicate VPM terminal arbors also have shorter rostrocaudal axes (~300 μms) than those in VPL. Whether trigeminal axons also produce multiple terminal arborizations along the rostrocaudal axis of a rod, like those in VPL, remains to be determined.

CO staining has been used to define CO-rich rods and CO-poor matrix in the monkey VPM. CO-rich rods were equated with the modality- and place-specific rods observed physiologically (Jones et al., 1982; Jones, 1983). These authors further described HRP terminal patches that seemed to match the dimensions of the CO-rich compartments (Rausell and Jones, 1991b). The CO-rich domains were reported to range from 100 to 1,000 μm in diameter in the coronal plane (Jones et al., 1986a; Rausell and Jones, 1991a,b). Our measurements of CO rods were smaller, ranging between 70 and 440 μm. Nevertheless, the BDA labeled terminal arbors demonstrated in the present study are smaller than all but the smallest CO-rich rod domains. Specifically, the mean terminal arbor diameters (86 ± 31 μm, contralateral, and 99 ± 26 μm, ipsilateral) that we obtained are dramatically smaller than the mean CO rod diameter (180 ± 87 μm). Direct comparison of the BDA labeled patches and the pattern of CO staining in the present study indicates that multiple axons arborize within a CO-rich rod. Thus, the larger diameter HRP labeled terminal patches seen in VPM (Rausell and Jones, 1991b) are likely to be made up of multiple pV terminal arbors. Recently, Cerkevich et al. (2013) combined VGluT immunohistochemistry and CO staining in an attempt to reveal a possible relationship between VGluT2 expression and CO labeling in VPM of macaque monkeys. They found VGluT2 densities within CO-rich rods, suggesting that VGlut2 reveals a finer anatomical subdivision of VPM. As neurons in pV are the source of the trigeminothalamic terminal arbors that are smaller than the CO-rich rods and are likely to be VGluT2 positive, they may correlate with these finer VGlut2 subdivisions.

### Relationship of Trigeminothalamic Arbors to Thalamic Neurons

The small mediolateral diameter of the thick fiber terminal arbors suggests that each may only influence a small number of target cells. Only a few counterstained somata were observed within an arbor. Thus, the small circumscribed arbors in VPM are well suited to play a role in maintaining the discrete topographic representation established at the periphery.

The dendritic fields of intracellularly stained VPM neurons have diameters ranging from 310 to 456 μm (cat: Ohara et al., 1995; macaque: Havton and Ohara, 1994; rat: Ohara and Havton, 1994). The present study has established that the diameters of terminal arbors within contralateral and ipsilateral VPM range up to 200 μm, with mean diameters of 86 ± 31 μm and 99 ± 26 μm, respectively. Clearly, the BDA labeled arbors in VPM are not of sufficient size to encompass a thalamic neuron’s dendritic tree. LM and EM examination of thick fiber terminal arbors suggest that a primary target of pV axons is proximal dendrites of VPM neurons. A similar arrangement for medial lemniscal axons was observed in the monkey VPL (Jones, 1983; see Figure 2B). These data are also in agreement with the EM findings in monkey VPL that show a near absence of somatic contacts on VPL somata, and a robust proximal dendritic distribution of medial lemniscal terminals (Ralston and Ralston, 1994). The present ultrastructural findings in VPM are very similar, although terminations on smaller diameter dendrites were also present. A recent study by Ge et al. (2010) demonstrated that pV trigeminothalamic axon terminals in the rat display vesicular glutamate transporters (VGluT1 or VGluT2 mRNA) when labeled by *in situ* hybridization. These axon terminals were shown to make asymmetric synapses on the dendritic profiles of VPM neurons. The similar terminals we observed are a good match for excitatory, glutamatergic transmission.

The proximal dendritic distribution shown here would put the pV axons in a position to act as a powerful synaptic drive for their target neurons. This effect would be enhanced by the high density of synaptic contacts on proximal dendrites found at both the LM and EM level in the present study. Thus, the organization of the thick fiber terminal arbors suggests that the macaque VPM is organized like other thalamic sensory nuclei where the large terminals of the ascending pathways act as “drivers” of thalamic neuron physiology (Sherman and Guillery, 1998; Bickford, 2016; Sherman, 2017). Indeed, the sizes of the pV boutons measured in our study are quite similar to those of the pV boutons reported in the mouse VPM that were assigned a driver role based on the physiological effects of their activation (Mo et al., 2017).

### Fiber Types

The present study identified two BDA labeled axon types that are differentially distributed in the primate VPM. They share some morphologic features with the cat terminal arbors described by Ericson et al. (1996) following injections of *Phaseolus vulgaris* leucoagglutinin into the marginal zone of the spinal trigeminal nucleus, pars caudalis. They placed arbors into three categories: “clusters”, “passing pearls”, or “passing beads”, based on their associated boutons. Our thick fibers with a variety of swellings resemble their “passing pearl” arbors. Our thin fibers with the small swellings resemble their “passing bead” type of termination. Veinante and Deschênes (1999) utilizing BDA injections in the rat, also labeled two types of trigeminothalamic terminal arbors arising from pV. Thick fibers arborized within a single VPM barreloid, while thin fibers were distributed to non-barreloid areas. A comparison with the present data indicates the predominant axon arbors in the rat and monkey VPM look qualitatively similar (Peschanski, 1984; Williams et al., 1994; Veinante and Deschênes, 1999; Veinante et al., 2000). One striking feature is the presence of large, irregularly shaped swellings associated with the trigeminothalamic axons in both species, suggesting that this may be a common mammalian feature.

Are there physiologic correlates to the observed fiber types? The information that is conveyed to pV from the face is mainly of the “lemniscal,” discriminative tactile type, and is carried by large, myelinated primary afferent fibers (Kerr et al., 1968). However, physiologic data exists suggesting some nociceptive signals are also present in pV (Azerad et al., 1982). Furthermore, there are polymodal cells in pV that respond to both noxious and non-noxious stimuli (Yu and King, 1974; Dallel et al., 1990). Veinante and Deschênes (1999) correlated the thick fibers with lemniscal-like, physiologically identified, single whisker sensitive neurons in pV. The thin fibers were associated with identified multiple whiskers sensitive neurons in pV. Sherman associated large boutons with driver inputs and small boutons with modulator inputs (Sherman and Guillery, 1998; Bickford, 2016; Sherman, 2017). Thus, it is reasonable to conclude that the thick fibers with large irregular boutons we observed are transmitting driver-type information from the face and oral cavity, while thin fibers with small boutons we observed are likely providing a modulator-type input. This would correlate well with the fact that the dominant feature we observed was thick fiber terminal arbors, and that thin fibers are relatively fewer in number. Also, the long course and widespread distribution of the thin fiber axons suggests that they are not transmitting detailed, topographically organized information.

## Data Availability Statement

The raw data supporting the conclusions of this article will be made available by the authors, without undue reservation.

## Ethics Statement

The animal study was reviewed and approved by Institutional Animal Care and Use Committee (IACUC)—University of Mississippi Medical Center Office of Animal Welfare.

## Author Contributions

DA and SW designed the experiment. DA, SW, and PM conducted the experiments and contributed to analyzing data and crafting the manuscript.

## Conflict of Interest

The authors declare that the research was conducted in the absence of any commercial or financial relationships that could be construed as a potential conflict of interest.

## Abbreviations

5n: trigeminal nerve
7n: facial nerve
At: axon terminal
At*: BDA labeled axon terminal
Ax: axon
BC: brachium conjuntivum
BDA: biotinylated dextran amine
CM: centromedian nucleus
CO: cytochrome oxidase
Den: dendrite
IC: inferior colliculus
MCP: middle cerebellar peduncle
mV: trigeminal motor nucleus
P: pyramid
PB: phosphate buffer
pc: parvocellular ventral posterior medial nucleus
PBN: parabrachial nuclei
PG: periaqueductal gray
Pul: pulvinar nucleus
PSD: presynaptic dendrite
pV: principal trigeminal nucleus
SC: superior colliculus
SO: superior olivary nuclues
VI: abducens nucleus
VII: facial motor nucleus
VPI: ventral posterior inferior nucleus
VPL: ventral posterior lateral nucleus
VPM: ventral posterior medial nucleus.
